# Torsion of Testis in an Infant with Unilateral UDT

**DOI:** 10.1155/2010/438530

**Published:** 2010-11-07

**Authors:** Mohammad Kazem Moslemi, Mehdi Abedinzadeh, Shabir Al-Mousawi

**Affiliations:** ^1^Department of Urology, Kamkar Hospital, School of Medicine, Qom University of Medical Science, Qom 3715694978, Iran; ^2^Department of Urology, Moradi Hospital, School of Medicine, Rafsanjan University of Medical Sciences, 3715694969 Rafsanjan, Iran; ^3^Division of Urology, Department of Surgery, Al-Amiri Hospital, 13041 Kuwait City, Kuwait

## Abstract

Torsion of an undescended testis is uncommon. Torsion of a cryptorchid testicle presents a nonspecific symptomatology. Clinical suspicion indicates emergent surgical exploration, irrespective of Doppler ultrasound with its inherent false negative results. Management of the contralateral testis is controversial. We emphasize the need of a complete physical examination of the child who goes to the emergency room with nonspecific symptoms of abdominal pain and ipsilateral empty hemiscrotum to rule out torsion of a cryptorchid testicle. Herein, we report a one-year-old infant with missed torsion of undescended left testis.

## 1. Case Presentation

**Figure 1:**
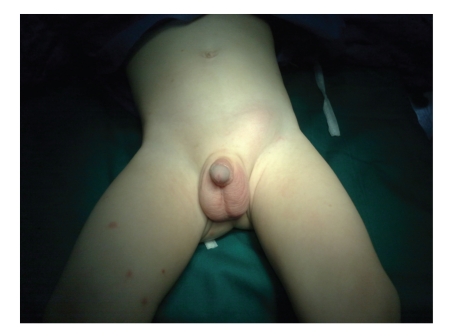
Swollen left inguinal area is evident.

**Figure 2:**
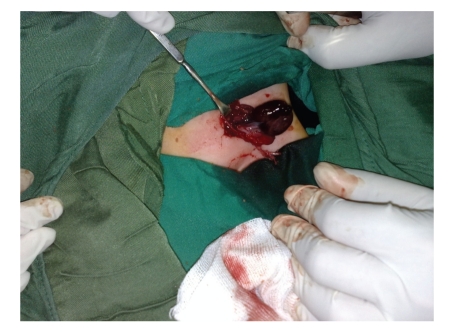
Necrotic testis with its overlying layers.

**Figure 3:**
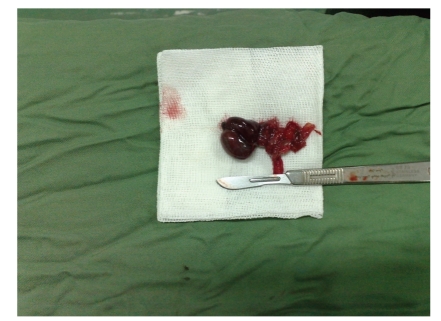
Removed necrosed testicle.

The patient was a one-year-old infant that presented with 3-day history of poor feeding, restlessness, progressive left inguinal swelling, and tenderness. In physical examination, a swollen left inguinal area with redness, point tenderness, and ipsilateral empty hemiscrotum was noted. The parents noted that they had been informed about the presence of a left UDT in their infant 3 months before. In the sonographic evaluation of testes, the right testis was normal, but the inguinal left testis was edematous, and no testicular circulation was found in Doppler evaluation of it. The patient underwent surgical exploration with left inguinal incision under the diagnosis of missed testicular torsion. After opening of layers, necrotic deep extravaginal testicular torsion that was located at the deep inguinal area was noted (Figure 1). Due to the complete necrosis of testicle, orchiectomy was performed (Figures 2 and 3). Surgical orchiopexy of the right side was postponed, because it was in normal position, and there is a risk of compromising its circulation due to manipulation or impending infection or parenchymal injury. 

## 2. Discussion

Cryptorchidism constitutes a common congenital surgical problem encountered in males in pediatric urology. The incidence of cryptorchidism or maldescended testis in full-term neonates is estimated to be from 2.7% to 5.9% at birth, but decreases to 1.2% or 1.8% by age 1 year [1]. The literature concerning undescended testis mainly concentrates on the increased risks of infertility and germ cell development as the primary sequel of this condition [2]. Yet, the UDT also appears to be at higher risk for torsion compared to the normally descended testis. Torsion of the spermatic cord was first described by Delasiauve in 1840, and interestingly, it was in a 15-year-old boy with the UDT treated with orchiectomy [3]. Williamson estimated torsion to be approximately 10 times more common in cryptorchidism [4]. Still, this issue is currently poorly addressed and sometimes neglected even in contemporary articles concerning pediatric testicular problems [1]. The mechanism of torsion in the undescended testis is not well understood. It has been postulated that it is related to a greater relative broadness of the testis compared to its mesentery [5], which is a possible explanation for the reported association with testicular tumors [6, 7]. Another theory involving abnormal contractions of the cremasteric muscles, which are responsible for the spermatic twist [8]. The diagnosis of torsion of an undescended testis should be considered in every child presenting with unexplained groin or abdominal pain in the presence of tender groin, swelling, and an empty ipsilateral hemiscrotum [5, 7]. Not unexpectedly, there is not enough awareness among physicians or parents with regard to this urological emergency, and in most cases, the diagnosis unfortunately is deferred. Testicular torsion (TT) accounts for only about 25% of all cases of acute scrotum in children [9], but requires prompt diagnosis and treatment in order to avoid ischemic necrosis of the testis. Some of the other differential diagnoses of acute scrotum in children's group are incarcerated hernia, torsion of testicular appendages, epididymoorchitis and trauma. Clinical problems and medicolegal issues are not limited to the preoperative diagnosis of testicular torsion (TT)—in normal descended or cryptorchid testis. In many cases, during exploration, the clinician faces the dilemma of whether to remove a testicle whose viability seems questionable and, in the absence of objective criteria, can only rely upon their own empirical experience [10]. Duration of symptoms before surgery is a well-known predictor of outcome in TT [11]. Another somewhat controversial point is whether prophylactic fixation of the contralateral testis is indicated [3, 8, 12]. While the reported salvage rate in testicular torsion ranges from 20% to 92% [8, 12], there are no such data available concerning undescended testis.

## 3. Conclusion

Torsion of an undescended testis is a relatively rare phenomenon that should be suspected, diagnosed, and treated without delay. With improved recognition of this entity and earlier referrals of patients with undescended testes by primary care physicians, this entity might eventually be prevented. As for the diagnosis of torsion in normal descended testis, there is no one history, physical, laboratory, or radiological finding that might predict testicular salvageability. This can only be determined at surgical exploration and is independent of the degree of torsion. In addition, when it is remembered that close to 2% of all males have an undescended testis, the risk of torsion in an undescended testis clearly needs to be made more evident to pediatric urologists and pediatricians.
